# Non-Substituted Imidazolium-Based Electrolytes as Potential Alternatives to the Conventional Acidic Electrolytes of Polyaniline-Based Electrode Materials for Supercapacitors

**DOI:** 10.3390/molecules29112569

**Published:** 2024-05-30

**Authors:** Fatima Al-Zohbi, Fouad Ghamouss, Johan Jacquemin, Bruno Schmaltz, Mohamad Fadel Tabcheh, Mohamed Abarbri, Khalil Cherry, François Tran-Van

**Affiliations:** 1 Department of Chemistry, Faculty of science III, Lebanese University, Tripoli 1300, Lebanon; mtabcheh@ul.edu.lb; 2 Materials Science and Nano-Engineering, Mohammed VI Polytechnic University, Lot 660 Hay Moulay Rachid, Ben Guerir 43150, Morocco; fouad.ghamouss@um6p.ma (F.G.); johan.jacquemin@um6p.ma (J.J.); 3 Laboratoire de Physico-Chimie des Matériaux et des Electrolytes pour l’Energie (EA 6299), Université de Tours, Parc de Grandmont, 37200 Tours, France; bruno.schmaltz@univ-tours.fr (B.S.); mohamed.abarbri@univ-tours.fr (M.A.); 4 Laboratoire Matériaux, Catalyse, Environnement et Méthodes Analytiques (MCEMA), Campus Universitaire de Hadath, Beirut 1500, Lebanon; khalil.cherry@ul.edu.lb

**Keywords:** polyaniline, protic ionic liquid-based electrolytes, Walden plot, electrochemical behaviors

## Abstract

Although disubstituted imidazolium cation is sterically crowded, hundreds of ionic liquids based on this cation have been reported as electrolytes for energy storage devices. In contrast to disubstituted imidazolium, non-substituted imidazolium is uncrowded sterically and has not yet been investigated as an electrolyte, to the best of our knowledge. Hence, imidazolium hydrogen sulfate [Imi][HSO_4_], in mixture with water, was studied as an electrolyte for PANI-based electrode materials. For comparison, pyrrolidinium with hydrogen sulfate or *p*-toluene sulfonate ([Pyrr][HSO_4_] or [Pyrr][PTS]), in mixture with water, were also investigated as alternatives to the conventional electrolyte (i.e., aqueous H_2_SO_4_) for PANI electrodes. Walden plots of binary mixture ionic liquid–water weight ratios with the optimal ionic conductivity (i.e., [Imi][HSO_4_]/water 48/52 wt% (195.1 mS/cm), [Pyrr][HSO_4_]/water 41/59 wt% (186.6 mS/cm), and [Pyrr][PTS]/water 48/52 wt% (43.4 mS/cm) along with the electrochemical performances of PANI in these binary mixtures showed that [Pyrr][HSO_4_]_aq_ or [Imi][HSO_4_]_aq_ are convenient electrolytes for PANI/PIL, as opposed to [Pyrr][PTS]_aq_. Furthermore, replacing the conventional aqueous electrolyte H_2_SO_4_ with [Imi][HSO_4_] _aq_ increased the specific capacitance of PANI/PIL from 249.8 to 268.5 F/g at 15 mV/s. Moreover, PANI/PIL electrodes displayed a quasi-ideal capacitive behavior in [Imi][HSO_4_]_aq_ (the correction factor of CPE_4_ was 0.99). This primary study has shown that non-substituted imidazolium as an electrolyte could enhance the electrochemical performances of PANI electrodes and could be a good alternative to the conventional electrolyte.

## 1. Introduction

Polyaniline (PANI) is one of the conducting polymers that have been considered as promising electrode materials for supercapacitors, owing to its properties such as thermal and electrochemical stability, high electronic conductivity in its doped state, low cost, and simple synthesis. Furthermore, the theoretical mass specific capacitance of PANI is relatively high: about 750 F/g for a potential range of 0.7 V and a maximum doping rate of 0.5 [1,2,3,4,5,6,7,8,9,10,11]. However, it has been found that the specific capacitance and the other electrochemical performances of PANI are widely dependent on the nature and the structure of the electrolytes [12,13,14,15].

The scientific community looks forward to replacing conventional electrolytes with ionic liquids in order to improve the electrochemical properties of energy storage devices such as supercapacitors or batteries [16]. Ionic liquids are promising candidates since they can display intrinsic stability and broad electrochemical windows depending on the nature of the selected ions (−2.5 to +2.5 V versus Ag/Ag^+^) [10,16]. Furthermore, they are characterized with negligible vapor pressure and good ionic conductivity [10]. 

Pairing PANI with ionic liquids seems an effective strategy to provide long-cycle-stable supercapacitors with high capacitance [10,17]. It is possible to categorize these combinations between PANI and ionic liquids into three approaches. The first approach (approach I) consists of focusing on the morphology of PANI alert by replacing the conventional acidic polymerization media with ionic liquids, followed by an electrochemical investigation of the obtained nanostructured PANI in the conventional electrolytes [3,18,19,20,21,22]. The second approach (approach II) concentrates on the electrochemical study of the PANI in the ionic liquid-based electrolytes [23,24,25]. The third approach (approach III) is established on the synchronization of the previously mentioned two approaches: synthesis of PANI in the ionic liquid and thus, electrochemical investigation of the resulted PANI in the same ionic liquid in which it was prepared [21]. 

Disubstituted imidazolium-based ionic liquids (i.e., aprotic ionic liquids, AILs) are the most investigated ionic liquids that have been used to evaluate the electrochemical performances of PANI [10]. This type of ionic liquid has also been investigated for other materials-based supercapacitors [16,26]. At the other extreme, non-substituted imidazolium-based ionic liquids, i.e., protic ionic liquids (PILs), to the best of our knowledge, have not yet been investigated as electrolytes for PANI, although they can be easily prepared with a neutralization reaction between imidazole and the corresponding acid. It is thus crucial to study the electrochemical performances of PANI following approach III using non-substituted imidazolium-based ionic liquid as electrolyte.

In our previous work, it was reported that PANI prepared in imidazolium hydrogen sulfate [Imi][HSO_4_] (i.e., non-substituted imidazolium salt) exhibited nanostructured morphology, enhanced electrical conductivity, and better electrochemical performance in conventional acidic electrolytes (following approach I) compared with the conventional PANI [18]. In the current work, the electrochemical performances of PANI prepared in [Imi][HSO_4_] was investigated in aqueous solution of [Imi][HSO_4_]. This study involved not only examination via approach III, but also analysis of the electrochemical performances of PANI in non-substituted imidazolium as electrolyte. For comparison, the electrochemical performances of PANI was also studied in pyrrolidinium with hydrogen sulfate or *p*-toluene sulfonate as electrolyte. Pyrrolidinium hydrogen sulfate was used as it has been reported as a convenient electrolyte for PANI [11].

## 2. Results and Discussion

This paper is divided into two main parts. In the first part, we present the synthesis and characterization of the investigated PILs. The other part shows the electrochemical performances of PANI/PIL electrodes in the selected PIL-based aqueous electrolytes.

**Part I: Synthesis and characterization of the protic ionic liquids**

The investigated PILs were imidazolium hydrogen sulfate [Imi][HSO_4_], pyrrolidinium hydrogen sulfate [Pyrr][HSO_4_], and pyrrolidinium *p*-toluene sulfonate [Pyrr][PTS]. They were prepared through simple acid–base reaction and dried under vacuum. The structures of these ionic liquids were studied using ^1^H NMR spectroscopy in deuterated dimethyl sulfoxide (CD_3_)_2_SO at room temperature (i.e., 20 °C); the obtained spectra are shown in Figures S1 and S2 in the Supporting Information. The chemical shifts (ppm) of the identified peaks are summarized in Table 1. It is noticeable that the chemical shift of the peak attributed to labile hydrogen of the H-N^+^ group (δ(N-H)) of imidazolium-based ionic liquids (5.5 ppm) was much lower than that of pyrrolidinium-based ionic liquids (8.53–8.55 ppm). Our team found that the δ(N-H) values of some PILs were related to the water content [27]. Herein, the difference between the δ(N-H) of the different PILs cannot be attributed to the water ratio as the residual water, quantified via Karl Fischer titration, was low and similar for all the selected PILs (i.e., 1.5, 1.8, and 2.7 wt% for neat [Imi][HSO_4_], [Pyrr][HSO_4_], and [Pyrr][PTS], respectively). On the other hand, Snook et al. [28] reported that δ(N-H) reflects the degree of proton activity (i.e., the concentration of protons) of electrolytes: δ(N-H) of about 7.05 ppm is an indication of highly acidic properties while δ(N-H) of 9.15 ppm designates a highly basic product. pH, estimated using an indicator paper, showed that [Imi][HSO_4_] was slightly more acidic than [Pyrr][HSO_4_] and [Pyrr][PTS] (all the estimated pH was below 1). As a result, the shift of δ(N-H) was not due to the acidity. Therefore, the difference between the δ(N-H) of imidazolium and pyrrolidinium was due to its electronic environment and its capability to interact with the anions. 

**Figure 1:**
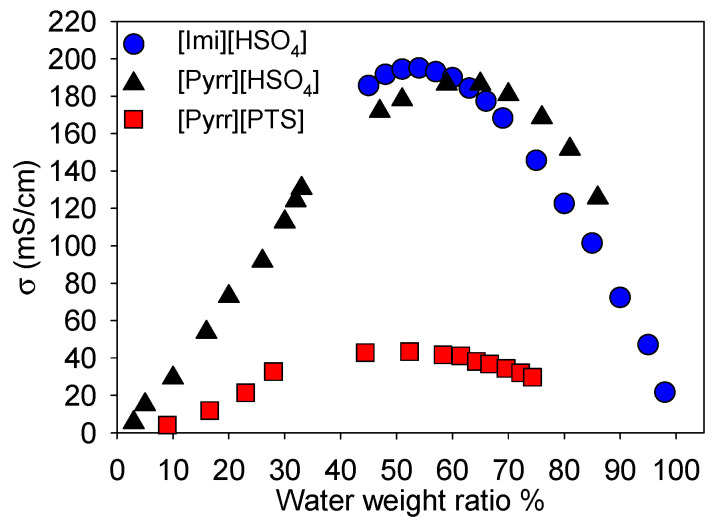
Ionic conductivity (σ) as a function of water mass ratio for [Imi][HSO_4_], [Pyrr][HSO_4_], and [Pyrr][PTS].

As the transport properties (ionic conductivity, viscosity) of the ionic liquids largely affect the electrochemical behaviors of PANI and significantly depend on the ionic liquid–solvent ratio [11,24,29,30,31,32,33], the ionic conductivities of the different investigated PILs were measured as a function of water weight ratio. Note that water was chosen as solvent because it is a green solvent and well adapted for electrochemical storage of PANI. Figure 1 presents the evolution of the ionic conductivity as a function of the PILs’ water weight ratio. From Figure 1, as expected based on the literature [27,33], it can be seen that the obtained ionic conductivity values gradually increased with the addition of water until an optimum, then progressively decreased for all the selected PILs. The ionic conductivity was relatively low for the selected PILs mixed with the minimum amount of water required to become liquid (i.e., the ionic conductivity was 8, 6, and 20 mS/cm for [Pyrr][HSO_4_] (1.5 wt% of water), [Pyrr][PTS] (10 wt% of water), and [Imi][HSO_4_] (20 wt% of water), respectively). The optimal ionic conductivity was obtained at different water weight ratio for each mixture (i.e., 54, 59, and 52% for [Imi][HSO_4_], [Pyrr][HSO_4_] and [Pyrr][PTS], respectively). This nonmonotonic behavior is rationalized by competition between the dissociation, concentration, and mobility of the charge carriers, and has previously been observed for several imidazolium-based aprotic ionic liquids upon addition of water or ethanol [34]. It was also noticeable that the optimal ionic conductivity of [Imi][HSO_4_] (195.1 mS/cm) was higher than that of [Pyrr][HSO_4_] (186.6 mS/cm). As for [Pyrr][PTS], its ionic conductivity (43.4 mS/cm) was the lowest. For an explanation of the order of the obtained optimal ionic conductivity of the PILs, the ions–ions interactions of these PILs in water were visualized by predicting the cation–anion Coulombic interactions, calculated using a conductor-like screening model for real solvent (COSMO-RS).

Table 2 presents the predicted surface charge distributions (i.e., an illustration of the surface polarity) and the sigma profiles of all the investigated ions. The polar regions of ions are coded in red and blue colors on the map of surface charge distribution. The red color, for example, reflects the region of positive polarization charge or a negative charge density (the polarization charge density and the charge distribution have opposite signs). In other words, the red region indicates the ability of the anion to accept protons or preferable interactions with proton donors. 

When [Pyrr][HSO_4_] was compared with [Pyrr][PTS], it was found that they differed from each other by their anion. For PTS^−^ (anion of [Pyrr][PTS]), the red color in the 3D molecular surface charge distribution map is located on the sulfonate group SO_3_^−^. The localization of the charge on SO_3_^−^ is due to the presence of the benzene ring, which is electron-donating via resonance. Regarding the surface of HSO_4_^−^, the red and dark blue colors over its whole surface indicate that the charge densities were distributed over almost its entire surface. The localization of the charge in the case of PTS^−^ indicates relatively stronger anion–cation Coulombic interactions for [Pyrr][PTS] compared with [Pyrr][HSO_4_], reflecting that [Pyrr][PTS] tends to form ion pairing more easily in solution. Concerning COSMO volume, this was more than twice the value for PTS^−^ (187 Å^3^) compared with HSO_4_^−^ (81 Å^3^), indicating that the mobility (strongly depended on the molecular weight and ion sizes [35]) of PTS^−^ would be lower than that of HSO_4_^−^ in solution. Based on the difference of the mobility between these two anions and knowing that the ionic conductivity of ionic liquids is related to the mobility of ions [35], one can predict that the ionic conductivity of [Pyrr][PTS] is lower than that of [Pyrr][HSO_4_].

As for [Imi][HSO_4_] vs. [Pyrr][HSO_4_], the difference between them is their cation. It was observed that the charge was more delocalized at the imidazolium cation (with the dark blue color delocalized between the nitrogen and the acidic proton) compared with the pyrrolidinium cation (with the blue color only on the nitrogen atom). Thus, imidazolium cation could exhibit relatively weaker Coulombic interactions with HSO_4_^−^ relative to pyrrolidinium cation, reflecting better ionic dissociation of [Imi][HSO_4_] in solution. Furthermore, the calculated COSMO volume of imidazolium (87 Å^3^) was much smaller than that calculated for pyrrolidinium (106 Å^3^), indicating better mobility of imidazolium.

[Imi][HSO_4_] was more dissociated in solution than [Pyrr][HSO_4_], which was in its turn better dissociated compared with [Pyrr][PTS]. The predicted order of the ionic conductivity was coherent and the obtained optimal ionic conductivity values of the selected PILs were similar (see Figure 1). 

[Imi][HSO_4_]/water 46/54 wt%, [Pyrr][HSO_4_]/water 41/59 wt%, and [Pyrr][PTS]/water 48/52 wt% (i.e., the binary mixtures of PILs/water with the optimum ionic conductivity), were selected to be used as electrolytes for PANI after investigation of their transport properties. Hereinafter, these selected binary mixtures are called [Imi][HSO_4_]_aq_, [Pyrr][HSO_4_]_aq_, and [Pyrr][PTS]_aq_. The selection of these mixtures is based on our previous work, where we showed that the electrochemical performances of PANI are related to the weight ratio of [Pyrr][HSO_4_]/water and the best results were obtained in [Pyrr][HSO_4_]/water with the optimal ionic conductivity.

**Figure 2:**
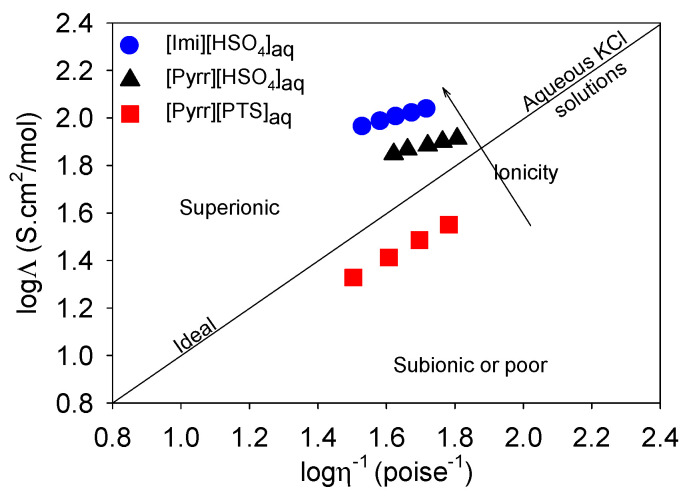
Walden plots for [Pyrr][PTS]_aq_, [Pyrr][HSO_4_]_aq_, and [Imi][HSO_4_]_aq_ (i.e., [Pyrr][PTS]/water 48/52, [Pyrr][HSO_4_]/water 41/59 and [Imi][HSO_4_]/water 48/52).

The transport properties (i.e., ionic conductivity, viscosity) as a function of temperature for the selected solutions of the PILs were measured and are presented in Figure S3 of Supporting Information. This data were used to establish the Walden plots, or more accurately, to gain an overview of the ionicity (i.e., the fraction of free ions) [36,37,38]. Figure 2 presents Walden plots of the selected binary mixtures of PILs/water between 30 and 50 °C. The molar conductivity Λ, presenting the ionic mobility, was calculated through division of ionic conductivity value σ (S/cm) by molar concentration (mol/cm^3^). The heavy black line (with a slope of unity) in Figure 2 represents the “ideal” Walden line, which means the absence of any ion–ion interactions. The ideal line is derived from dilute aqueous KCl or LiCl solutions (i.e., fully dissociated ionic solutions). Based on the position of ionic solutions against ideal line, they can be divided into three main classes [39]: subionic or poor (in the area below the ideal line), ideal (on the ideal line), and superionic solutions (in the area above the ideal line). For the mobility of ions, sometimes the classical Walden rule (Λη = constant, i.e., the diffusion of ions is controlled through the macroscopic viscosity of the electrolyte.) is not applicable, and thus, it is replaced by the ‘fractional’ Walden rule (Λη^γ^ = constant where 0 < γ < 1; i.e., decoupling of ion motions from viscosity) [37,38,39,40,41,42,43,44].

Like most ionic liquids and other binary mixtures of ionic liquids/water [39,40,41,42,43,44], the Walden plots of all the investigated electrolytes exhibited a slope (γ) below one, indicating some weak decoupling of ion motions from viscosity in this temperature range. For [Pyrr][PTS]_aq_, the Walden plot points were located below the ideal Walden line (subionic region), confirming the strong ionic pairing of PTS^−^ with pyrrolidinium. Indeed, Brauer et al. reported that ionic liquid with PTS^−^ as counter anion exhibited the poorest ionicity relative to [I], [NO_3_], [OMs], [BF_4_], [OTf] and [NTf_2_] accompanied by the strongest cation–anion interactions with triazolium cationic species [45]. When comparing [Pyrr][HSO_4_]_aq_ with [Imi][HSO_4_]_aq_, it was observed that the points for each were located in the upper part of the Walden diagram, indicating their good ionic behavior. The behavior of [Imi][HSO_4_]_aq_ tended more towards the superionic regime in comparison with [Pyrr][HSO_4_]_aq_. Imidazolium cation Imi^+^ provided ionic liquid with better mobility compared with pyrrolidinium cation Pyrr^+^, which may be attributed either to the difference between their size (COSMO volume 87 and 106 Å^3^ for imidazolium and pyrrolidinium, respectively) or, according to the predicted surface polarity, to the better ionic dissociation of [Imi][HSO_4_] in solution compared with [Pyrr][HSO_4_]. 

**Figure 3:**
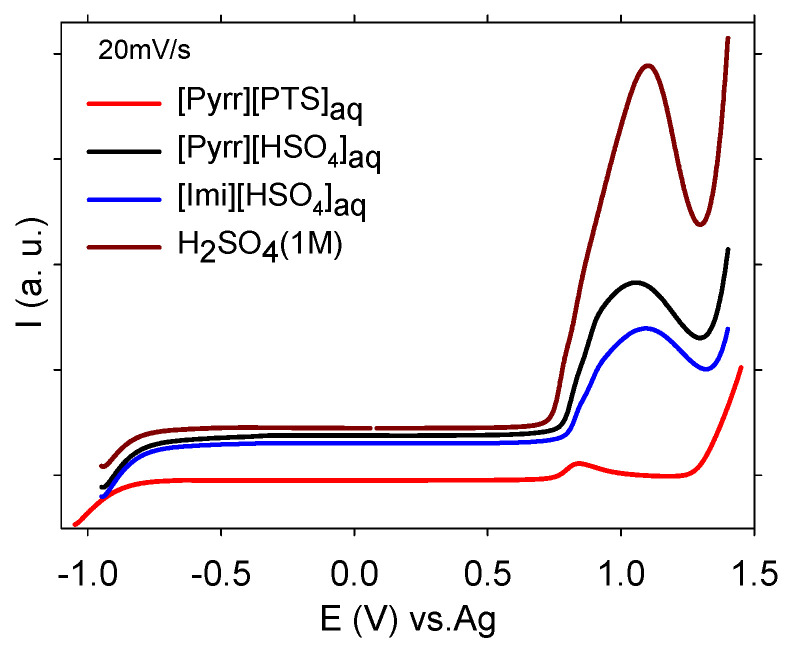
Electrochemical window at 20 mV/s of [Imi][HSO_4_]_aq_, [Pyrr][HSO_4_]_aq_, [Pyrr][PTS]_aq_, and H_2_SO_4_ 1 mol/L (working electrode and counter electrode: stainless still grid, reference electrode: Ag wire).

The electrochemical windows of the selected PILs were also studied in order to examine their resistance to the oxidation and reduction. It is well known that the electrochemical window of the electrolytes is a key parameter for electrochemical devices and a large window helps to prevent the side reaction and degradation of doped conjugated polymer [23]. The measurement was recorded via cyclic voltammetry at 20 mV/s in a steel grid (used as courant collector for evaluating the electrochemical behavior of PANI, as described in the next section), while the pseudo-reference electrode was Ag wire. Figure 3 shows the electrochemical stability windows for the investigated electrolytes as well as that of the conventional electrolyte for PANI (H_2_SO_4_ 1 mol/L). In oxidation, the hydrogen sulfate anions (the counter anion of [Pyrr][HSO_4_]_aq_, [Imi][HSO_4_]_aq_, and H_2_SO_4_) were oxidized at more positive potential than *p*-toluene sulfonate (the counter anion of [Pyrr][PTS]_aq_). According to Anouti et al. [46], the hydrogen sulfate anion is oxidized giving the persulfate (i.e., 2HSO_4_^−^ → H_2_S_2_O_8_ + 2ē). A peak was also observed located before that of the oxidation of hydrogen sulfate anions. That peak was attributed to oxidation of water (i.e., H_2_O → 1/2O_2_ + 2H^+^ +2ē). However, in reduction, the pyrrolidinium cation is deprotonated, followed by proton reduction to hydrogen gas (i.e., Pyrr^+^ + ē → Pyrr + H^+^; H^+^ + ē → 1/2H_2_) [46]. It was also noticeable that the electrochemical windows of the PILs were almost equal to that obtained for H_2_SO_4_. One can conclude that despite the addition of water into the investigated PILs, its electrochemical window remained suitable for examination of electrochemical behaviors of PANI.

**Part II: Electrochemical performances of PANI**

**Figure 4:**
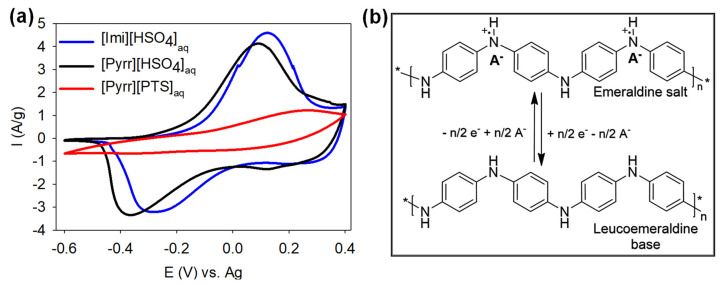
(**a**) CV curves at 5 mV/s for PANI in [Pyrr][PTS]_aq_, [Pyrr][HSO_4_]_aq_, and [Imi][HSO_4_]_aq_ and (**b**) redox process of PANI where n is the number of aniline units and A^−^ is the counter anion (A^−^: HSO_4_^−^ or PTS^−^).

The electrochemical performances of PANI/PIL were studied in a three-electrode system configuration using the three different kinds of PIL-based aqueous electrolytes: [Pyrr][PTS]_aq_, [Pyrr][HSO_4_]_aq_, and [Imi][HSO_4_]_aq_, presented in part I. Figure 4a shows the obtained CV curves of PANI/PIL at 5 mV/s in the investigated electrolytes. One can see that the CV curves resulted in [Imi][HSO_4_]_aq_ and [Pyrr][HSO_4_]_aq_ each presenting a redox couple, whose potential varied with different PILs being used. This redox couple is typically observed for PANI characterized in the conventional aqueous electrolytes [11,19]. It is associated with the reversible transformation between leucoemeraldine base (semiconducting state) and emeraldine salt (conducting state) forms [23,47,48,49,50]. The structures of these two oxidation states of PANI are shown in Figure 4b. Briefly, an electron is lost from the dimer aniline rings in the PANI, and thus, counterions diffuse from electrolytes to the PANI surface in order to compensate the created positive charge [23,47]. Regarding the CV curve of PANI/PIL in [Pyrr][PTS]_aq_, it displayed a weak anodic peak but no cathodic peak even at a very low scan rate (see Figure 4a and Figure S4 of the Supporting Information). The absence of a reduction peak could indicate the difficulty of the release of PTS^−^ out of the polymer, perhaps due to the large size of PTS^−^ (with a COSMO volume of about 187 Å^3^) or because [Pyrr][PTS]_aq_, according to Walden plot in Figure 2, is considered as a poor electrolyte (low dissociation). R. Pauliukaite et al. showed that an increase in solution pH caused a larger separation of the redox couple and a decrease in the peak currents, until no peak was observed in the potential region studied at pH 3.2, which was attributed to the loss of PANI conductivity due to deprotonation of the polyaniline at higher solution pH [47]. 

**Figure 5:**
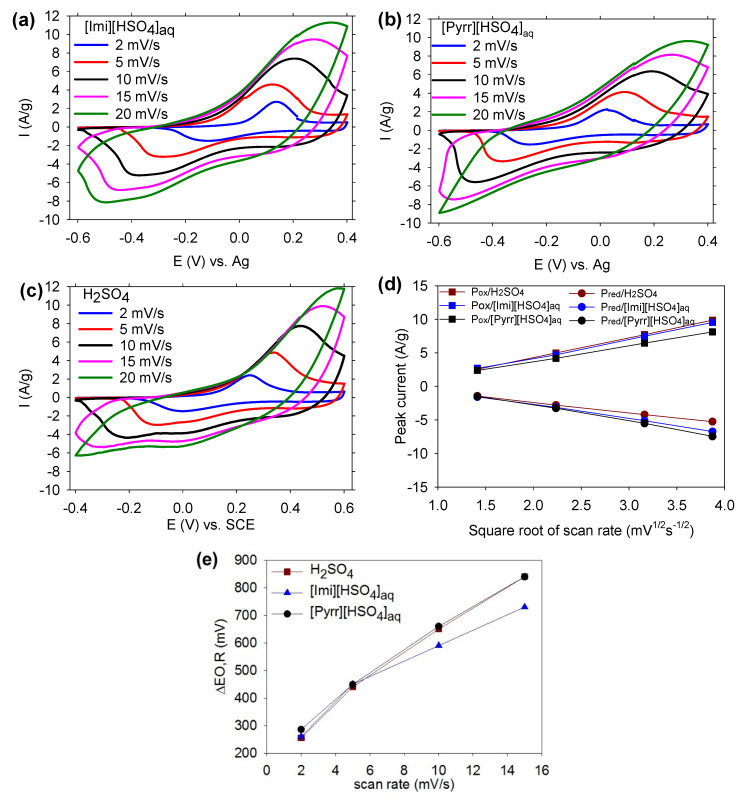
CV curves of PANI/PIL at different scan rates of 2, 5, 10, 15, and 20 mV/s in (**a**) [Imi][HSO_4_]_aq_, (**b**) [Pyrr][HSO_4_]_aq_, and (**c**) H_2_SO_4_; (**d**) the dependence of the anodic peak current (Ipox) and cathodic peak current (Ipred) current on the square root of the scan rate for PANI/PIL; and (**e**) plot of ΔE_O,R_ versus scan rate.

Figure 5a–c show the CV curves of PANI/PIL measured over 1 V in [Imi][HSO_4_]_aq_, [Pyrr][HSO_4_]_aq_, or H_2_SO_4_ 1 mol/L at a scan rate of 2, 5, 10, 15, and 20 mV/s. All the CV profiles, obtained at 2, 5, 10, and 15 mV/s, show the redox couple of PANI. However, the reversibility of this couple as well as the peak current depend not only on the electrolyte used but also on the scan rates. It was also noticeable that the cathodic peak disappeared in H_2_SO_4_ or [Pyrr][HSO_4_]_aq_ at 20 mV/s, while this was not the case in [Imi][HSO_4_]_aq_. Its disappearance was attributed to the charge transfer and diffusion that mainly occurred not inside the bulk but on the surface of the electrode [47,51].

Regarding the peak current of PANI/PIL, this increased along with the increment of scan rate, indicating the good rate capability of the PANI/PIL electrode. Figure 5d shows the currents of the redox peaks as a function of the square root of scan rate in all the electrolytes used, within the range 2–15 mV/s. The peak current value, according to the Randles–Sevcik equation, is related to the square root of the scan rate and the diffusion coefficient of the electroactive material [24,52]. The Randles–Sevcik equation is presented as follows:

(1) ip=2.69×105n32AD012v12c

where A is the electrode area (cm^2^), D is the diffusion coefficient (cm^2^/s) of counter ions, V is the scan rate (V/s), C_b_ is the concentration of electroactive center (mol/cm^3^), and I_p_ is the peak current (A). Since the peak current is linearly dependent on the root of the scan rate in these electrolytes, one can conclude, based on Equation (1), that the redox reaction of PANI/PIL is controlled by a diffusion process. It was also noticeable that the slope of each obtained line was different. These differences are likely to have been due to the transport properties of the electrolytes, as all the investigated electrolytes shared the same type of counter ion (HSO_4_^−^) and thus, the same affinity to PANI.

When the scan rate increased from 2 to 20 mV/s, the reduction and oxidation peaks shifted in negative and positive directions, respectively. This shift was due to the resistance of the electrode and some kinetic irreversibility [53,54]. The ΔE_O,R_ (i.e., the average of the potential of the anodic and the cathodic peaks) of the redox couple was calculated and is illustrated in Figure 5e as a function of the scan rate. At very low scan rates (i.e., 2 or 5 mV/s), ΔE_O,R_ was almost similar in all the electrolytes investigated. However, at scan rates of 10 or 15 mV/s, the effect of electrolytes on the ΔE_O,R_ of PANI/PIL was pronounced. The lowest ΔE_O,R_ was displayed in [Imi][HSO_4_]_aq_, indicating more reversibility of the PANI redox couple in [Imi][HSO_4_]_aq_, and therefore, faster kinetics of ion diffusion during the redox reaction. It has been shown that more reversibility of the redox couple of PANI may be associated with a higher Walden product (Λη) of the electrolyte [11]. The Walden product of [Imi][HSO_4_]_aq_ (2.7 S·cm^2^·mol^−1^·poise at 30 °C) was higher than that of [Pyrr][HSO_4_]_aq_ (1.6 S·cm^2^·mol^−1^·poise at 30 °C), which can explain the greater reversibility of the redox couple of PANI in [Imi][HSO_4_]_aq_.

The values of the specific capacitance were calculated from the discharge curves based on the mass of active materials using the following equation (Equation (2)):

(2) $$C=\frac{\int Idt}{m\times \Delta V}$$

where *C* is the specific capacitance of the active materials of both electrodes (F/g), *I* is the constant discharge current (mA), *dt* is the discharge time (s), Δ*V* is the voltage difference in discharge (V), and *m* is the total mass of the active material of both electrodes (mg). Obtained capacitance values vs. scan rate are summarized in Table 3. The specific capacitance decreased along with the increment of the scan rate, whichever electrolyte was used. Nevertheless, the decrement rate of the specific capacitance of PANI was related to the nature of the electrolyte. In H_2_SO_4_, a significant decrease of specific capacitance with the increment of scan rate was observed: the capacitance retention was only 89.1% when the scan rate increased from 5 to 15 mV/s. However, in [Imi][HSO_4_]_aq_, the specific capacitance slightly decreased when the scan rate increased from 5 to 15 mV/s, indicating a good retention of capacitance (96.1%). It was noted that the specific capacitance of PANI/PIL in the PIL-based electrolytes was higher than in H_2_SO_4_ at a scan rate of 15 mV/s. 

**Figure 6:**
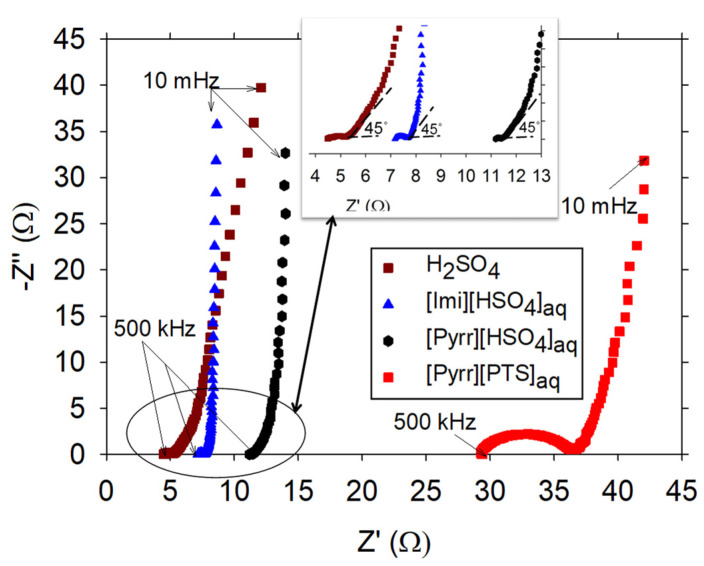
Nyquist plot at open circuit potential with AC amplitude of 10 mV over the frequency range from 500 KHz to 10 mHz.

Electrochemical behaviors of PANI/PIL were also characterized via electrochemical impedance spectroscopy (EIS) in order to evaluate the properties of charge transport in the PANI/PIL electrodes–electrolyte interface. Figure 6 shows the obtained Nyquist plots at open circuit potential for PANI/PIL electrodes in the different aqueous solutions of the PILs as well as in H_2_SO_4_. All obtained plots displayed a semi-circle followed by a section at 45°, rather than a straight line that is typically observed for PANI electrodes [55,56,57,58]. The semi-circle, located in the high frequency range, was attributed to the charge transfer. In the middle frequency region, the section at 45° was correlated to the Warburg resistance resulting from ion diffusion/transport in the electrolyte to the electrode surface. In the low frequency region, a straight line associated with ion diffusion in the electrode pores was observed. A brief comparison of all the obtained Nyquist plots is given as follows:

- At high frequency region (500 kHz), the intercept of impedance plot with the real axis gives the equivalent series resistance (R_1_), which represents the sum of the electrolyte solution resistance, the intrinsic resistance of active material, and the contact resistance at the electrode–electrolyte interface [55]. The R_1_ of PANI/PIL was close to 4.61, 6.86, 10.8, and 29.20 Ω in H_2_SO_4_, [Imi][HSO_4_]_aq_, [Pyrr][HSO_4_]_aq_, and [Pyrr][PTS]_aq_, which have ionic conductivity values of 237 (this value has been adapted from reference [59]), 195, 186, and 43 mS/cm, respectively. The value of R_1_ decreased along with the increasing ionic conductivity of the electrolyte. The dependence of R_1_ on the transport properties of the electrolyte is coherent with what has been reported in the literature [24,60].
- The semi-circle is due to a parallel RC element: an interfacial charge–transfer resistance R_2_ and double-layer capacitance [61]. R_2_ can be estimated from the diameter of the semi-circle. Since the semi-circle obtained for [Pyrr][PTS]_aq_ was much wider that those resulting from in the other investigated electrolytes, the R_2_ of PANI/PIL in [Pyrr][PTS]_aq_ was thus significantly higher relative to the others, indicating bad interface properties between the electrode and [Pyrr][PTS]_aq_. This observation is in accord with the CV curves of Figure 4a, where it is shown that [Pyrr][PTS]_aq_ was a poor electrolyte for PANI. As for the two other PIL-based electrolytes ([Pyrr][HSO_4_]_aq_ or [Imi][HSO_4_]_aq_), the diameter of semi-circle was relatively smaller (associated with lower R_2_) than that obtained with H_2_SO_4_ (see the inset of Figure 6), revealing a good contact between electrode and electrolyte.
- Warburg behavior (i.e., the frequency dependence of the ion diffusion/transport in the electrolyte [62]) was less pronounced in [Imi][HSO_4_]_aq_ than the other investigated electrolytes, indicating the capacitive behavior of PANI/PIL in [Imi][HSO_4_]_aq_.
- In the low frequency region, PANI/PIL displayed a more vertical line with [Pyrr][HSO_4_]_aq_ than H_2_SO_4_, while the line obtained with [Imi][HSO_4_]_aq_ was almost parallel to the imaginary axis. It is known that a line parallel to the imaginary axis reveals a short ion diffusion path and a more ideal capacitor, which means efficient electrolyte accessibility to the electrode surface [63,64].

**Figure 7:**
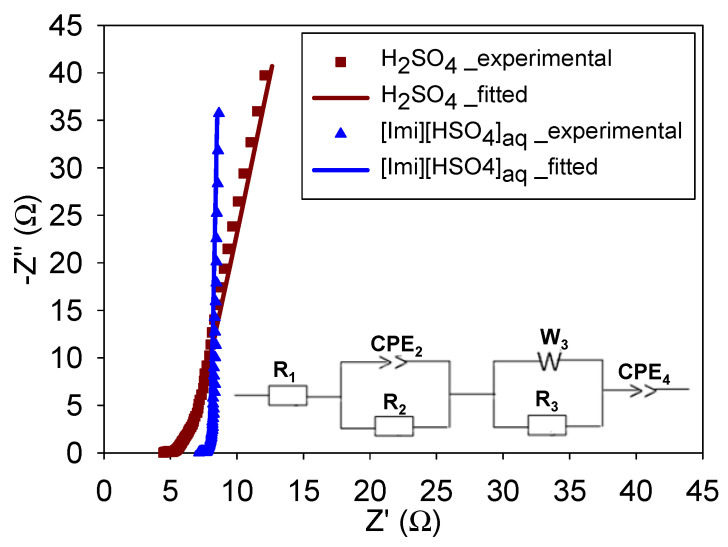
Nyquist plot of PANI/PIL electrodes in H_2_SO_4_ or [Imi][HSO_4_]_aq_; symbols and lines denote experimental and fitted data, respectively. The inset is the equivalent circuit model.

The EIS data obtained for H_2_SO_4_ and [Imi][HSO_4_]_aq_ were simulated in order to obtain quantitative information. Figure 7 shows the experimental data as well as the fitted model, which was obtained using the equivalent circuit shown in the inset in Figure 7. This model, proposed by Ferloni et al. [58], includes the following elements: the equivalent series resistance (R_1_), the electrical double-layer capacitance at the electrolyte–PANI interface (CPE_2_), the charge transfer resistance (R_2_), the Warburg impedance (W_3_), the ionic diffusion resistance of the polymer (R_3_), and the pseudocapacitance (CPE_4_). Note that CPE is the constant phase element, which is used instead of a pure capacitance due to the non-ideal behavior and related to an empirical constant (α) ranging from 0 to 1 in the following equations (Equations (3) and (4)) [58,65,66]:

(3) $$Z_{CPE}=A_{CPE}(j\omega {)}^{-n}$$

(4) $$Z_{CPE}=T_{CPE}\;(j\omega {)}_{CPE}^{-\alpha }$$

where *T* and *α* are frequency-independent constants and ω is angular frequency.

Table 4 lists the obtained EIS fitting values. When H_2_SO_4_ was replaced with [Imi][HSO_4_]_aq_, the R_2_ of the PANI/PIL electrode decreased from 0.58 to 0.42 Ω. Similarly, R_3_ fell from 1.71 to 0.78 Ω. Note also that the Warburg coefficient for [Imi][HSO_4_]_aq_ (0.89 Ω·s^−1/2^) was smaller than that of H_2_SO_4_ (1.92 Ω·s^−1/2^). Regarding the constant phase element, the CPE_4_ of PANI/PIL in [Imi][HSO_4_]_aq_ showed an improvement compared with that obtained in H_2_SO_4_, and as a result, its estimated correction factor α_4_ (0.99) was close to that of an ideal capacitor (α = 1).

## 3. Materials and Methods

### 3.1. Materials

Aniline from Acros was distilled under reduced pressure before use. Sulfuric acid (95–98%, Alfa Aesar, Ward Hill, MA, USA), *p*-toluene sulfonic acid (98.5%, Sigma Aldrich, St. Quentin Fallavier, France), ammonium peroxodisulfate (98%, Sigma Aldrich, St. Quentin Fallavier, France), pyrrolidine (≥99%, Fluka, Buchs, Switzerland), imidazole (≥99.5%, Sigma Aldrich, St. Quentin Fallavier, France), activated carbon (Super DLC-50, Norit, Amersfoort, Utrecht, The Netherlands), carbon black (super C65, Timcal, Congleton, UK), and polytetrafluoroethylene (60%wt dispersion in H_2_O, Aldrich, St. Quentin Fallavier, France) were used as received. 

### 3.2. Synthesis of the Investigated Ionic Liquids 

All the investigated ionic liquids (i.e., pyrrolidinium hydrogen sulfate [Pyrr][HSO_4_], pyrrolidinum *p*-toluene sulfonate [Pyrr][PTS], and imidazolium hydrogen sulfate [Imi][HSO_4_]) were synthesized through equimolar acid–base reactions according to the procedure described in previous articles [11,67]. The synthesis was carried out using a three-necked round-bottomed flask immersed in an ice bath and equipped with a reflux condenser, a dropping funnel, and a thermometer. Pyrrolidine or imidazole was introduced into the three-necked round-bottomed flask while the corresponding acid (sulfuric acid or *p*-toluene sulfonic acid) was added dropwise, through the dropping funnel, to the base under vigorous stirring. The reaction temperature was kept below 20 °C using an ice bath. After finishing the addition of the acid, the reaction mixture was stirred for 30 min at 20 °C and then, the ice bath was removed. The mixture was then stirred overnight at room temperature. The resulting product was then dried for 24 h under primary vacuum. The collected [Imi][HSO_4_], [Pyrr][HSO_4_], or [Pyrr][PTS] was a dark brown solid, a yellow oily liquid, or a white solid, respectively, at room temperature. The molecular formulas and molecular mass of [Imi][HSO_4_], [Pyrr][HSO_4_], and [Pyrr][PTS] are C_3_H_5_N_2_.HSO_4_ (166.2 g/mol), C_4_H_8_NH_2_.HSO_4_ (169.1 g/mol), and C_4_H_8_NH_2_.C_7_H_7_SO_3_ (243.1 g/mol), respectively. Melting points, estimated via Kofler hot bench, are 61 and 67 °C for [Imi][HSO_4_], and [Pyrr][PTS], respectively. [Pyrr][HSO_4_] is viscous at room temperature.

### 3.3. Synthesis of the PANI 

PANI/PIL was prepared as described in our previous work [18]: 58.9 g of [Imi][HSO_4_] was added to anilinium hydrogen sulfate solution (3 g in 18 mL of water) under stirring at 5 °C. Then, the solution of ammonium peroxodisulfate (NH_4_)_2_S_2_O_8_ (4.56 g in 10 mL of water), cooled to 5 °C, was slowly added to anilinium solution. The oxidant (NH_4_)_2_S_2_O_8_ to aniline mole ratio was 1.25/1 and the [Imi][HSO_4_] to water weight ratio in the final mixture was 70/30. The final obtained mixture was stirred for 24 h at 5 °C with the use of a thermostat. Then, a solid product was isolated via filtration, intensely washed with water to pH neutral, then dried under vacuum at 60 °C for 12 h. Green powder was obtained.

The formation of PANI/PIL was confirmed using attenuated total reflection infrared (ATR-IR) spectroscopy. The collected spectrum, described in our previous work [18], showed peaks at 1544 cm^−1^ (stretching of quinone ring N=Q=N), 1390 cm^−1^ (stretching of benzene rings N-B-N), 1286 cm^−1^ (C-N stretching), 916 cm^−1^ (C–H in-plane deformation), and 728 cm^−1^ (C-H out-of-plane bending vibration). Regarding the morphology of the obtained PANI/PIL, the scanning electron microscopy SEM images presented in our previous work [18] show that PANI/PIL exhibits a fibrillar homogeneous morphology.

### 3.4. Measurements 

The ^1^H spectrum was recorded using a Varian Unity Inova 300 MHz at room temperature. The data are given as chemical shifts in δ (ppm). (CH_3_)_4_Si (TMS, 0 ppm) was used as an internal standard.

Residual water content was determined using coulometric Karl Fischer titration (Metrohm, 831 KF coulometer). 

Viscosity (η) measurements were conducted using an Anton Paar Lovis 2000 M/ME rolling-ball viscometer. The temperature in the cell was regulated to within ±0.02 °C. The viscosity standard (ASTM Oil Standard S600 of CANNON, 1053 mPa s at 25 °C) and ultra-pure water were used to calibrate the viscometer. In this study, the uncertainty of reported viscosity measurements did not exceed ±1%. 

Conductivity measurements were performed using a biologic instrument as a function of temperature from 5 to 70 °C. Temperature control was ensured to within ±0.01 °C by means of a JULABO thermostated bath. The conductometer was first calibrated with standard solutions of known conductivity (i.e., (0.1 and 0.02) mol/dm^3^ KCl aqueous solutions). Each conductivity was recorded when its stability was better than 1% within 2 min, and the uncertainty of reported conductivities did not exceed ±2%.

### 3.5. Electrode Preparation and Electrochemical Measurements

All electrochemistry measurements were carried out on a versatile multichannel potentiostat (Biologic S.A) piloted with the EC Lab V10.32 interface at room temperature (i.e., 20 °C), using a classical three-electrode configuration cell, as illustrated in Figure S5. For the working electrode (WE), a paste was first prepared by mixing 60 wt% of active material (PANI), 32 wt% of carbon black, and 8 wt% of polytetrafluoroethylene (PTFE) in a minimal amount of ethanol using a pestle and mortar to make a homogeneous paste. The obtained paste was then laminated on a glass surface prior to being dried to remove the volatile solvent used. Each WE was then fabricated via pressing the dried paste on stainless steel mesh under 10 tons pressure. The typical mass of paste loading on each WE was close to 1.5 mg/cm^2^. Similarly, the counter electrode (CE) was prepared through pressing 7 mg of a paste containing 70 wt% of activated carbon, 20 wt% of carbon black, and 10 wt% of PTFE on a stainless steel grid under 10 tons pressure. The electrolytes used were aqueous H_2_SO_4_ (1 mol/L) (i.e., the conventional acidic electrolyte for PANI) or aqueous solutions of the investigated protic ionic liquids (i.e., [Imi][HSO_4_]/water 46/54 wt%, [Pyrr][HSO_4_]/water 41/59 wt%, or [Pyrr][PTS]/water 48/52 wt%).

The cyclic voltammetry was carried out using the previously described three-electrode cell with saturated calomel electrode SCE, with KCl as reference electrode when aqueous H_2_SO_4_ (1 mol/L) was the electrolyte, while an Ag wire was used as a pseudo reference electrode for an aqueous solution of protic ionic liquid-based electrolyte. No potential correction was needed, as the main goal of this preliminary work was to study the reversibility of the PANI.

For the electrochemical impedance spectroscopy (EIS) measurements, the previously mentioned electrochemical set-up was prepared and the reference electrode was saturated calomel electrode for all the investigated aqueous electrolytes (i.e., H_2_SO_4_ (1 mol/L), [Imi][HSO_4_]/water 46/54 wt%, [Pyrr][HSO_4_]/water 41/59 wt%, or [Pyrr][PTS]/water 48/52 wt%).

### 3.6. Computational Methods

First, the structure of each of the studied ions was optimized in the gas phase with a convergence criterion of 10^−8^ Hartree, through DFT calculations combining the resolution of identity (RI) approximation [68,69] within the Turbomole 7.0 program package [70], using the B3LYP function with the def-TZVP basis set [71,72,73]. The resulting optimized structures were then used as inputs in the COSMOconfX program (version 4.0, using the DFT/BP-86/def-TZVP COSMO and GAS calculation options available) to generate the conformers of each species, which were then able to be used in the COSMOthermX software (version C30 17.05, mixture option) to determine the lowest energy contact between two identical clusters. The COSMO volume and the sigma profile of each ion were then generated using the COSMO-RS (conductor-like screening model for real solvent) methodology within the COSMOthermX program (version 2.1, release 01.08).

## 4. Conclusions

Protic ionic liquids (PILs), namely [Imi][HSO_4_], [Pyrr][HSO_4_], and [Pyrr][PTS], were investigated as alternatives to the conventional acidic aqueous electrolytes (e.g., H_2_O/H_2_SO_4_) for PANI-based electrodes for supercapacitors. For that purpose, the ionic conductivity of each PIL was optimized by mixing it with water, and thus, the resulted binary mixtures with optimal ionic conductivity were tested as electrolytes using a three-electrode system configuration. The investigated electrolytes were [Imi][HSO_4_]/water 48/52 wt%, [Pyrr][HSO_4_]/water 41/59 wt%, and [Pyrr][PTS]/water 48/52 wt%, denoted as [Imi][HSO_4_]_aq_, [Pyrr][HSO_4_]_aq_, and [Pyrr][PTS]_aq_, respectively. This work draws the following conclusions:

- Reversibility of the redox couple of PANI is greatly dependent on the structure of the ionic liquids used as electrolyte. For [Pyrr][PTS]_aq_, no cathodic peak was observed on the CV curve of PANI even at very low scan rates, revealing that the anion of [Pyrr][PTS] was hardly released from the polymer during the reduction process. In [Pyrr][HSO_4_]_aq_ and [Imi][HSO_4_]_aq_, both the anodic and cathodic peaks of PANI were well defined. However, the redox couple of PANI was more reversible in [Imi][HSO_4_]_aq_ relative to [Pyrr][HSO_4_]_aq_ (i.e., ΔE_O,R_ of PANI was about 730 mV at 15 mV/s in [Imi][HSO_4_]_aq_, lower than that in [Pyrr][HSO_4_]_aq_ (with ΔE_O,R_ of about 840 mV at 15 mV/s).
- Approach III (presented in the introduction) led to more electrochemically performant PANI electrode materials than approach I. For example, the capacitance retention of the investigated PANI was about 89.1 and 96.1% in H_2_SO_4_ and [Imi][HSO_4_]_aq_, respectively, when the scan rate increased from 5 to 15 mV/s. However, this preliminary conclusion needs to be proven since at this stage it is not possible to deduce whether the enhanced electrochemical performances of PANI in [Imi][HSO_4_]_aq_ were due to its better physicochemical properties compared with the other investigated ionic liquids or to the approach followed.
- Non-substituted imidazolium-based ionic liquids or non-substituted pyrrolidinium-based ionic liquids could be good alternatives to the mono- or disubstituted imidazolium salts widely investigated in the literature as electrolytes for storage devices.

## Figures and Tables

**Table 1:** ^1^H NMR spectrum characteristics of the PILs.

Structure of the selected PILs	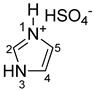 [Imi][HSO_4_]	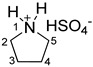 [Pyrr][HSO_4_]	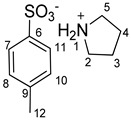 [Pyrr][PTS]
δ(N-H) ppm	5.5 (broad singlet, 1H, H_1_)	8.53 (broad singlet, 2H, H_1_),	8.55 (broad singlet, 2H, H_1_)
δ(C-H) ppm	8.9 (s, 1H, H_2_) 7.6 (d, 2H, *J* = 1.2, H_5_, H_4_)	3.09–3.07 (m, 4H, H_2_, H_5_) 1.81–1.77 (m, 4H, H_3_, H_4_)	7.54–7.44 (m, 2H, H_7_ and H_11_) 7.13 (d, *J* = 8.0 Hz, 2H, H_8_, H_10_) 2.98–3.18 (m, 4H, H_2_, H_5_) 2.29 (s, 3H, H_12_) 1.87–1.76 (m, 4H, H_3_, H_4_)

**Table 2:** 3D molecular surface charge distributions, COSMO volume, surface, and sigma profiles for the ions of the investigated ionic liquids.

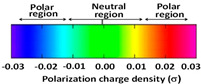	COSMO volume (Å^3^)	Surface (Å^2^)	Sigma profiles
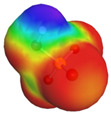 Hydrogen sulfate (HSO_4_^−^)	81	98	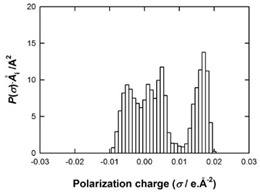
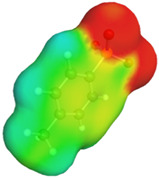 *p*-toluene sulfonate (PTS^−^)	187	187	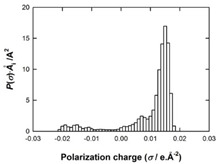
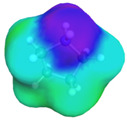 Pyrrolidinium (Pyrr^+^)	106	117	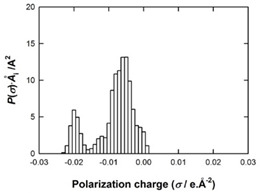
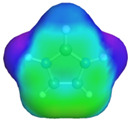 Imidazolium (Imi^+^)	88	103	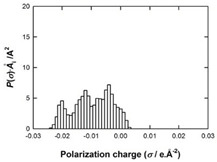

**Table 3:** Specific capacitance of PANI/PIL at different scan rates.

Electrolyte	Capacitance (F/g) at Varying Scan Rate (mV/s)			Capacitance Retention (%)
	5	10	15	100 × (C_15_/C_5_)
H_2_SO_4_	280	272	250	89.1
[Imi][HSO_4_]_aq_	279	276	268	96.1
[Pyrr][HSO_4_]_aq_	290	285	265	91.4

**Table 4:** The resulting fitting values of the Nyquist plot of PANI/PIL electrode in H_2_SO_4_ or [Imi][HSO_4_]_aq_.

Electrolytes	R_1_ (Ω)	R_2_ (Ω)	CPE_2_ (F·s^a−1^)	α_2_ (Ω)	R_3_ (Ω)	σ_3_ (Ω·s^−1/2^)	CPE_4_ (F·s^a−1^)	α_4_
[Imi][HSO_4_]_aq_	6.86	0.42	1.01 × 10^−3^	0.79	0.78	0.89	0.44	0.99
H_2_SO_4_	4.61	0.58	2.88 × 10^−3^	0.67	1.71	1.92	0.3	0.91

## Data Availability

Data are contained within the article and Supplementary Materials.

## Supplementary Materials

The following supporting information can be downloaded at: https://www.mdpi.com/article/10.3390/molecules29112569/s1.
