# Supplementary material for: Non-Substituted Imidazolium-Based Electrolytes as Potential Alternatives to the Conventional Acidic Electrolytes of Polyaniline-Based Electrode Materials for Supercapacitors
Source: Molecules. 2024 May 30;29(11):2569. doi: 10.3390/molecules29112569 (PMC11173517; doi:10.3390/molecules29112569)
Supplement: Supplementary file 1 [file molecules-29-02569-s001.zip › molecules-2981186-supplementary.pdf]

# Electronic Supporting Information

# Non-Substituted Imidazolium-Based Electrolytes as Potential Alternatives to the Conventional Acidic Electrolytes of Polyaniline-Based Electrode Materials for Supercapacitors

**Fatima Al-Zohbi [1,*], Fouad Ghamouss [2], Johan Jacquemin [2], Bruno Schmaltz [3], Mohamad Fadel Tabcheh [1], Mohamed Abarbri [3], Khalil Cherry [4] and François Tran-Van [3,*]**

[1] Department of Chemistry, Faculty of science III, Lebanese University, Tripoli 1300, Lebanon; mtabcheh@ul.edu.lb

[2] Materials Science and Nano-Engineering, Mohammed VI Polytechnic University, Lot 660 Hay Moulay Rachid, Ben Guerir 43150, Morocco; fouad.ghamouss@um6p.ma (F.G.); johan.jacquemin@um6p.ma (J.J.)

[3] Laboratoire de Physico-Chimie des Matériaux et des Electrolytes pour l'Energie (EA 6299), Université de Tours, Parc de Grandmont, 37200 Tours, France; bruno.schmaltz@univ-tours.fr (B.S.); mohamed.abarbri@univ-tours.fr (M.A.)

[4] Laboratoire Matériaux, Catalyse, Environnement et Méthodes Analytiques (MCEMA), Campus Universitaire de Hadath, Beirut 1500, Lebanon; khalil.cherry@ul.edu.lb

* Correspondence: alzohbi-fatima@hotmail.com (F.A.-Z.); francois.tran@univ-tours.fr (F.T.-V.)

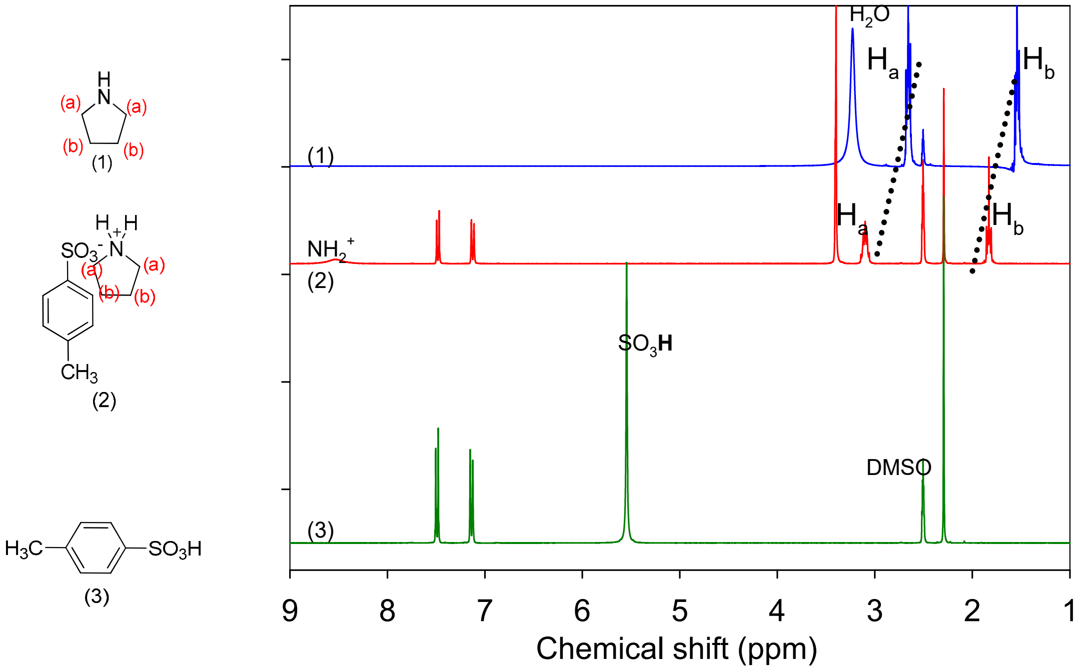


**Figure S1** : $^{1}H$ NMR spectra of (1) pyrrolidine, (2) [Pyrr][PTS] and (3) *p*-toluenesulfonic acid in deuterated DMSO

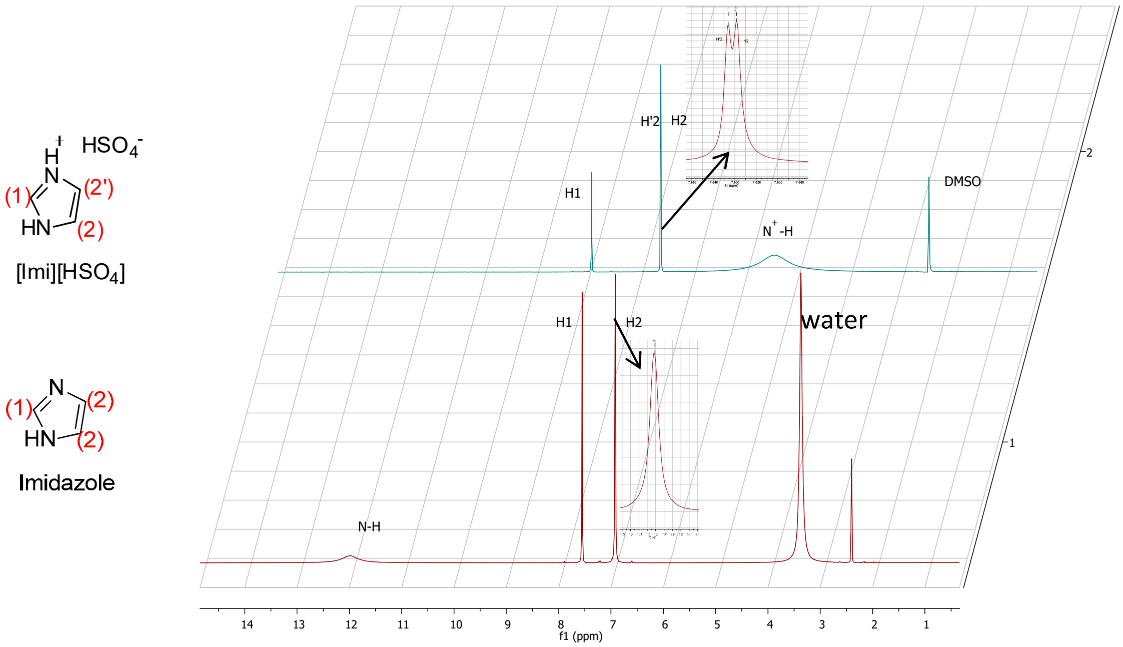


**Figure S2** : $^{1}H$ NMR spectra of [Imi][$HSO_4$] and its starting reagent : Imidazole in deuterated DMSO (copyright, applied functional materials 2022)

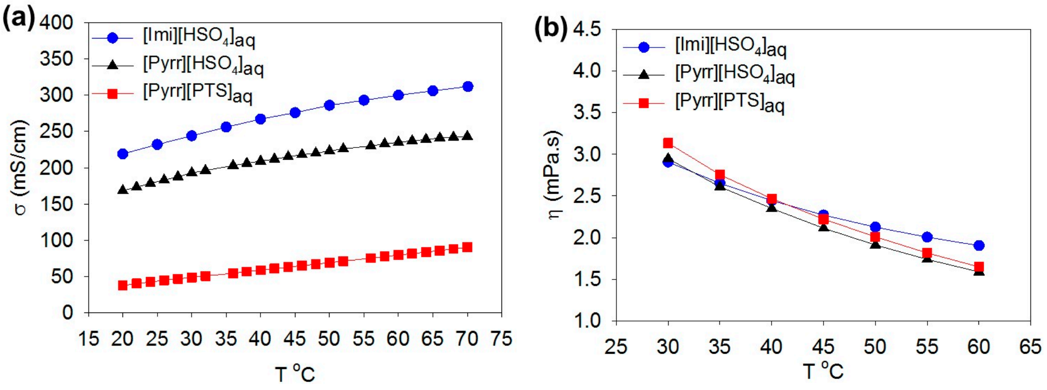


**Figure S3** : (a) Viscosity and (b) ionic conductivity values as a function of temperature for [Imi][$HSO_4$]$_{aq}$, [Pyrr][$HSO_4$]$_{aq}$ and [Pyrr][PTS]$_{aq}$ (*i.e.* [Imi][$HSO_4$]/water 48/52 wt%, [Pyrr][$HSO_4$]/water 41/59 wt% and [Pyrr][PTS]/water 48/52 wt%)

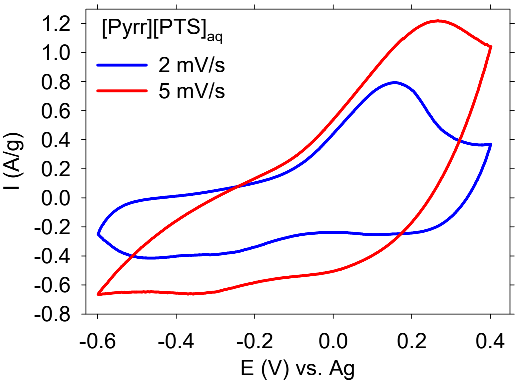


**Figure S4** : CV curves of PANI/PIL at different scan rates of 2 and 5 mV/s in [Pyrr][PTS]$_{aq}$

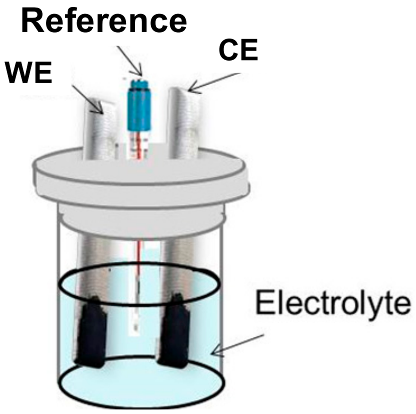


**Figure S5** : Schematic representation of the three-electrode cell used to perform the electrochemical characterization (WE: working electrode et CE: counter-electrode, reference electrode is saturated calomel electrode); WE is near the reference electrode and close to the CE, the cell should be transparent to watch if the color of the electrolyte change during the electrochemical test.
